# Occupational Exposure during the Production and the Spray Deposition of Graphene Nanoplatelets-Based Polymeric Coatings

**DOI:** 10.3390/nano13081378

**Published:** 2023-04-15

**Authors:** Irene Bellagamba, Fabio Boccuni, Riccardo Ferrante, Francesca Tombolini, Claudio Natale, Fabrizio Marra, Maria Sabrina Sarto, Sergio Iavicoli

**Affiliations:** 1 Research Center for Nanotechnology Applied to Engineering (CNIS), Sapienza University of Rome, I-00185 Rome, Italy; 2 Department of Astronautical, Electrical and Energy Engineering (DIAEE), Sapienza University of Rome, I-00184 Rome, Italy; 3 Italian Workers’ Compensation Authority—Department of Occupational and Environmental Medicine, Epidemiology and Hygiene, Via Fontana Candida 1, I-00078 Rome, Italy; 4 Directorate General for Communication and European and International Relations, Italian Ministry of Health, Lungotevere Ripa 1, I-00153 Rome, Italy

**Keywords:** nanotechnologies, smart materials, nanostructured coating, occupational safety and health, risk mitigation

## Abstract

Graphene-based polymer composites are innovative materials which have recently found wide application in many industrial sectors thanks to the combination of their enhanced properties. The production of such materials at the nanoscale and their handling in combination with other materials introduce growing concerns regarding workers’ exposure to nano-sized materials. The present study aims to evaluate the nanomaterials emissions during the work phases required to produce an innovative graphene-based polymer coating made of a water-based polyurethane paint filled with graphene nanoplatelets (GNPs) and deposited via the spray casting technique. For this purpose, a multi-metric exposure measurement strategy was adopted in accordance with the harmonized tiered approach published by the Organization for Economic Co-operation and Development (OECD). As a result, potential GNPs release has been indicated near the operator in a restricted area not involving other workers. The ventilated hood inside the production laboratory guarantees a rapid reduction of particle number concentration levels, limiting the exposure time. Such findings allowed us to identify the work phases of the production process with a high risk of exposure by inhalation to GNPs and to define proper risk mitigation strategies.

## 1. Introduction

In recent years, the state of the art of carbon-based nanomaterials (NMs) has undergone considerable development thanks to research in processes and products, as well as to scientific, technological, and experimental discoveries which made them particularly suitable for use as nanofillers in polymer matrices for the development of innovative multifunctional and smart materials. They find wide application in different industrial fields such as wearable electronics [1,2,3,4,5], smart textiles [6], structural sensing and monitoring [7,8,9], and electromagnetic interference (EMI) solutions [10,11,12]. Among them, graphene-based polymer composites have been widely studied thanks to the combination of lightness, easy workability, and cost-effectiveness properties. Moreover, they have unique features, such as extremely high electrical conductivity, mechanical strength, and thermal stability [13,14]. The use of these innovative systems involves the production of materials at the nanoscale and their handling in combination with other materials.

Some occupational studies have shown that the introduction of these materials into the workplace can cause adverse unpredictable effects and potentially health concerns for exposed workers [15,16]; therefore, particular attention should be paid to their safe use and production.

An overview of the literature has shown that although graphene-based NMs hazard assessment is coming of age, with increasing numbers of studies addressing the potential impact of these materials on living systems, data gaps still remain [17]. Furthermore, the unusual aerodynamic behavior of platelet-like graphene structures has raised concerns regarding the exposure by inhalation [18].

At present, no official occupational exposure limits (OELs) for graphene are available [19], even though Lee et al. [20] proposed an OEL of 18 μg/m^3^ for graphene, based on subchronic inhalation studies in rats. Although a nano reference value 8 h time-weighted average (NRV_8 h TWA_) of 40,000 part/cm^3^ for particles having density lower than 6 g/cm^3^ was proposed [21], no NRVs are recommended for non-spheroidal NMs, such as graphene-based materials [22]. Furthermore, for peaks lasting a few seconds, a NRV 15 min time-weighted average (NRV_15 min TWA_) calculated as two times the NRV_8 h TWA_, was proposed by van Broekhuizen et al. [23] as considerable support for risk assessment of NPs.

Limited data are also available on airborne graphene-based material concentrations in occupational settings [24]. Spinazzé et al. [25] studied the exposure in the production of graphene family NMs by a multi-metric direct reading approach, highlighting that workers directly involved in material sampling for quality control have a high potential for occupational exposure. In the production of Few Layered Graphene (FLG), the powder storage and the equipment cleaning were the tasks with high particle concentration values, producing airborne nanostructures with the same morphology of FLG in the workplace [26,27]. Lee et al. [28] showed airborne graphene-like structures during the weighing and sonication operation of Graphene Nanoplatelets (GNPs), despite particle concentration values being similar to the background ones. GNP-like materials were also found in the workplace air during the weighing of powder, the addition of liquid, and mixing [29].

Due to the lack of knowledge on NMs exposure data, the U.S. National Institute for Occupational Safety and Health (NIOSH) has proposed the implementation of the Prevention-through-Design (PtD) approach as a framework aimed at preventing risks and protecting workers, starting from the design stage of the work cycle [30,31]. The PtD criteria can be applied to safely design engineered NMs and to optimize their production process, use, and manipulation with the aim of eliminating or minimizing the related risks [32].

In the present study, the PtD principles have been applied to the NMs emissions during the design and production of graphene-based materials for sensor applications with the aim of identifying the process steps with high potential risk for workers and proposing recommendations to set up proper risk mitigation strategies. In the previous study conducted by the same authors [33], workers’ exposure during the production process of GNPs by liquid exfoliation in the Research and Development (R&D) labs was evaluated. The worst exposure conditions were identified during the thermal expansion phase near the operator and in the process performed at the highest temperature.

In this framework, the aim of the present study is to extend the NMs emissions measurements to the work phases after the GNPs production; this involves the realization of an innovative nanocomposite coating with controlled electrical and electromagnetic properties. The coating, made of a water-based polyurethane paint filled with GNPs, is deposited by spray casting technique for producing multifunctional piezoresistive materials for both electromagnetic and Structural Health Monitoring (SHM) applications [34].

The exposure measurements were conducted by using a multi-metric approach—one of the consensus methods adopted throughout the recently published literature [35,36,37]—and follow the harmonized tiered approach published by the Organization for Economic Cooperation and Development (OECD) [38,39]. This approach allows the evaluation of multiple exposure data collected by using a combination of direct reading measurement methods and morphological and elemental analysis of airborne sampled materials.

## 2. Materials and Methods

### 2.1. Production Process of the GNP-Filled Polyurethane Composite

**Figure 1:**
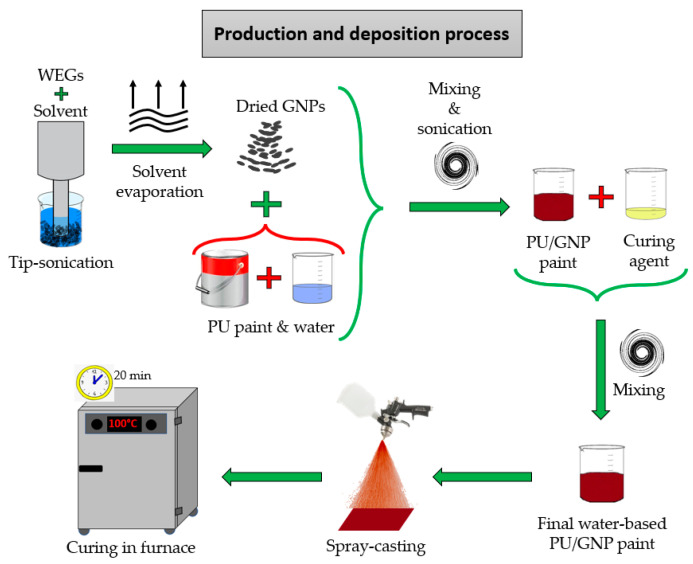
Schematic representation of the production and deposition process of the nanostructured coating.

The production process of the graphene-based composite, sketched in Figure 1, was described in Fortunato et al. [8] and it consisted of the following steps. At first, GNPs were produced by thermal expansion as already described in Bellagamba et al. [33] and Sarto et al. [40] and tip sonicated in acetone. The GNP-acetone suspension was then placed in the furnace to permit the complete evaporation of the solvent, obtaining a fine GNP dry powder. A polyurethane (PU) paint was added to the dried GNPs and mixed with deionized water (DI). To promote a good dispersion of GNPs inside the paint and to avoid their agglomeration or aggregation, the mixture was homogenized by the following tree mixing steps: mechanical blending, high shear mixing, and tip-sonication. A proper amount of curing agent was then added to the nanostructured paint and mixed with a mechanical stirrer.

The final composite was spray-coated with an airbrush over a polycarbonate (PC) substrate and then cured in a furnace at 100 °C for 20 min.

The manufacturing process has been designed for the production of two different types of nanocomposite materials: a paint loaded with 3.5 %wt of GNPs (paint Type A) was produced for developing strain sensors for SHM applications; while the second one, filled with 8 %wt of GNP (paint Type B), was used for producing Radar Absorbing Materials (RAM) for low radar observability applications.

The production process of both types of nanostructured paints is usually carried out by a single worker, and it can be schematically divided into the following two phases:

- Phase 1: tip-sonication of the worm-like expanded graphite (WEG)/acetone suspension and solvent evaporation in hoven to obtain a GNP dried powder;
- Phase 2: mixing and sonication steps of dried GNPs with PU paint, DI water, and curing agent; spray casting deposition with airbrush of the final coating.

Phase 1 is identical for both production processes, except for the quantity of GNPs necessary to produce each nanostructured paint. For this reason, the tip sonication in acetone was conducted only once, by using the total quantity of the material needed for the production of both types of paints. Meanwhile, phase 2 of the production process (mixing and spray deposition) was executed separately for each paint.

The deposition of the coating by spray casting method can be performed in two ways, and in particular, the coating with lower GNPs concentration is generally sprayed by using an airbrush equipped with a nozzle of 0.8 mm, smaller than the nozzle used for the spray deposition of the coating with a GNPs’ concentration of 8 %wt, whose diameter is 1.2 mm. However, each paint was sprayed twice—the first deposition was performed with the smaller nozzle (0.8 mm) and the second deposition with the larger one (1.2 mm)—for a comparison between the two methodologies. Each spray deposition lasted 3 s.

The different quantities of each material used for the production of the two types of nanostructured paints are reported in Table 1.

### 2.2. Morphological and Chemical Characterizations of the Nanocomposite Coating

The morphological characterization of the produced nanocomposite was performed by Scanning Electron Microscopy (SEM, Zeiss, Oberkochen, Germany) on the nanostructured coating loaded with 3.5 %wt of GNPs and deposited by spray-casting on a metal substrate.

**Figure 2:**
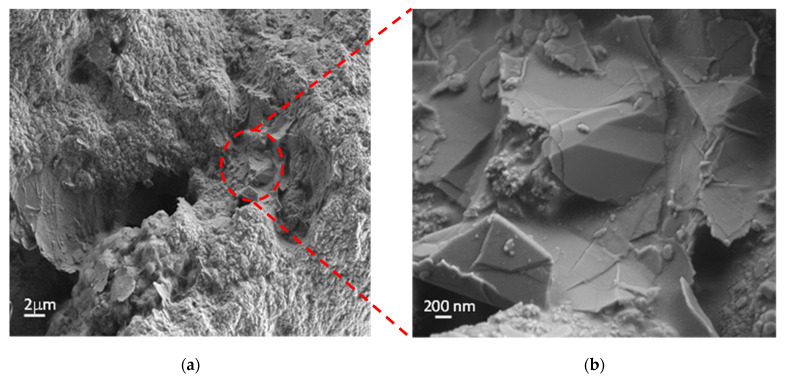
(**a**) SEM images of the top surface of the sprayed PU/GNP paint; (**b**) a detail of some GNPs dispersed in the PU matrix.

From SEM images reported in Figure 2, representing the top surface of the nanostructured coating (Figure 2a) (GNPs dispersed in the polyurethane matrix (zoom area in Figure 2b)), we can observe that the nanostructures are incorporated and well distributed within the matrix. We can recognize the typical geometry of GNPs, characterized by an irregular planar shape with sharp edges and wedges [40,41] and an average surface area of a few µm^2^ [41].

### 2.3. Workplace Description

The production process took place in a laboratory of area of about 20 m^2^ located on the ground floor of a 3-story building of the Sapienza University of Rome. The production laboratory has a natural ventilation system formed by a window of 2 m × 2 m, a mechanical ventilation system for air delivery equipped with M5 (EN779:2012) ePM10 65% (ISO 16890) filters, and an air conditioner for maintaining the lab at a constant temperature of ~21 °C. Windows remained closed in the lab during the production and were opened at the end of the whole process. Air exchange is managed electronically through a commercial sensor that determines the air quality of the room, which is taken from the building’s terrace. It should be noted that the production room is a chemical laboratory in which materials are produced daily, and chemicals (i.e., organic solvents) are generally used and stored. Inside the lab, there is a chemical hood under which the tip-sonication (phase 1) is performed and a ventilated fume hood where the paint preparation and the spray deposition (phase 2) are performed. The furnace is located next to the fume hood. The chemical hood is equipped with a high-efficiency impeller with inverted blades, a Ø 225 inlet/outlet duct, and a flow rate with a range between 1000–2700 m^3^/h. The fume hood has a modular filtration column with HEPA filters GF4AS, according to the standards AFNOR NF X 15-211:2009 and BS. The maximum flow rate is 460 m^3^/h. The system is not hermetically sealed but it has been modified so that the air velocity inside is greater than 2 m/s. Both the hoods have a proper air extraction system, connected to the aspiration line of exhaust fumes.

Personal protective equipment (PPE) used by workers inside the lab includes laboratory coats, protective nitrile gloves, cold resistant gloves, and full-face respirators (mod. 6000 series, 3M^TM^, Beirut, Lebanon) equipped with filters (mod. 3M, 6099 ABEK2 P3 series) in accordance with the regulation UNI EN 14387.

Another room, in which no NMs were produced and no chemicals were used and stored, was selected for the simultaneous background measurements (see Section 2.5). This room is located next to the production laboratory, and it has same orientation, structural, and ventilation properties (a natural ventilation system formed by the window and a mechanical air delivery system equipped with M5 ePM10 65% filters) as the laboratory, except for the air conditioner that it is not present. The door of both the laboratory and the background room opens onto an internal corridor, while all the windows open to the outside of the facility.

### 2.4. Measurements and Sampling Method

The exposure to GNPs during the production phases of the two nanostructured paints has been assessed by adopting the same measurement strategy already applied to the case of the production of GNPs by thermal exfoliation. [18] We employed easy-to-use and hand-held instruments for real-time measurements and personal samplers for off-line analysis according to the tier 2 of the harmonized OECD measurement approach [38,39] as also recommended by the World Health Organization NMs guidelines [42].

In particular, the set of instruments used in the present study includes:

- Condensation Particle Counter (CPC mod. 3007, TSI Inc., Shoreview, MN, USA). It is an optical counter that can measure the Particle Number Concentration (PNC [part/cm^3^]) with a time resolution of 1 s and an accuracy of ±20%. CPC can detect nano-objects with an average size in the range between 10 and 1000 nm. CPC has a concentration range from 0 to 100,000 part/cm^3^ as declared by the manufacturer. The working fluid is Isopropanol;
- Mini Diffusion Size Classifier (DiSCmini, mod. TESTO, TESTO SE & Co. KGaA, Titisee-Neustadt, Germany). It is a diffusion charging (DC) instrument that measures three parameters—PNC (part/cm^3^), modal average diameter (D_avg_ [nm]), and Lung Deposited Surface Area (LDSA [µm^2^/cm^3^])—in the environment and in correspondence with the worker’s personal breathing zone (PBZ) with a time resolution of 1 s and an accuracy of ±30%. This instrument is able to detect airborne particles characterized by a diameter ranging from 10 nm to 700 nm for PNC measurements and from 10 nm to 300 nm for D_avg_ measurements. Sampling Tygon^TM^ tubes 1.5 m length have been used for DiSCmini (DM) measurements;
- Nanoparticle Surface Area Monitor (NSAM mod. 3550, TSI Inc., Shoreview, MN, USA). This instrument measures the average and cumulative LDSA (µm^2^/cm^3^) of particles from 10 nm to 1000 nm with 1 s time resolution, corresponding to the tracheobronchial (TB) or alveolar (A) pulmonary fractions, and based on the model published by the International Commission on Radiological Protection [43];
- Personal impactor (mod. Sioutas, SKC Inc., Eighty Four, PA, USA) equipped with 5 different filter stages. It separates and collects ultrafine, fine, and >2.5 µm airborne particles characterized by different aerodynamic diameters ranges: <0.25 µm, 0.25–0.50 µm, 0.50–1.0 µm, 1.0–2.5 µm, >2.5 µm (up to 10 µm). Particles above each cut point are collected on a 25 mm aluminum filter in each appropriate stage when the Sioutas is used with a 9 L/min sample pump. Particles of less than 0.25 µm cut point of the last stage are collected on a 37 mm PTFE after-filter.

For a comprehensive characterization of the exposure scenario, off-line morphological and elemental analysis of sampled materials collected by Sioutas during the production of the nanostructured paints has been conducted by High-Resolution Field Emission Scanning Electron Microscope (FE-SEM, Zeiss, Zeiss, Oberkochen, Germany) equipped with an Energy Dispersive X-ray Spectroscopy (EDS, Oxford Instruments INCA, High Wycombe, UK). The same analyses have been also performed on the trial samples materials in order to characterize GNPs shapes and distinguish them in the workplace air. This step is essential for the correct interpretation of data gathered during the real-time measurements and for their correlation with the findings of off-line analyses. The comparison between morphological and chemical analyses on airborne sampled materials in the workplace and the GNPs trial samples characterization may allow the confirmation of NMs emission during the production process of the PU/GNP paint.

The assessment of GNPs emission related to the manufacturing process has been carried out by comparing the PNC values measured during the production of the two paints (Type A and Type B) with the corresponding significant values that represent the threshold beyond which NMs emission by production may be supposed. According to the OECD tiered approach, PNC significant values have been calculated as the average background plus three times the standard deviation [38,44].

In order to characterize the indoor background, two different measurements were conducted [45]:

- Inside the production laboratory, before starting the manufacturing process, for Near-Field (NF) background characterization. The NF background measurements sessions lasted 15 min and they were conducted with the furnace off and the fume hood turned on, representing the standard conditions in the lab before starting the production process;
- In the other room not influenced by the process, where NMs are not produced/handled and no other sources of nanoparticles are present, simultaneously with the manufacturing process, for Far-Field (FF) background characterization.

Real-time data have been represented by time series and box-plot diagrams. The last ones represent a synthetic way to demonstrate the statistical distribution of a given time series. In the box-plot diagrams related to this case study, we have described the maximum and the minimum recorded value indicated by the highest whisker and the lowest whisker of each box. The line inside the box represents the median value, the square represents the mean value, and the upper and the lower edges of the box represent the 75th and the 25th percentile, respectively.

Polynomial curve fitting methods were also used to obtain the residual curve of PNC by subtracting the FF background contribution. The FF polynomial fit curve has been calculated and subtracted from the time series of the PNC measured inside the laboratory in order to obtain the PNC (NF and PBZ) residual curves during the production process phase 1.

All data analyses were performed using Origin Pro 2018 software, version b9.5.1.195 (Northampton, MA, USA).

### 2.5. Experimental Campaign Setting Up

Real-time measurements and sampling were conducted within three days as set out in the time sheet of Table 2. As already mentioned in Section 2.2, since phase 1 of the manufacturing process is the same for both types of paints, during the production of the paint type B, we only assessed phase 2, i.e., the mixing and sonication steps of the water-based paint with a higher amount of dried GNP, and the spray-coating deposition of the final PU/GNP paint (see Table 1). On day 1, preliminary measurements and instrument comparison sessions were performed; on day 2, the measurements were conducted before starting the production process, during phase 1 and during the production phase 2 of the paint type A (phase 2A); on day 3, the measurements were performed during the production phase 2 of the paint Type B (phase 2B). At the end of the measurement campaign (day 3), another instrument comparison session (parallel session) was conducted.

Preliminary measurements and samplings in the lab when no activities occurred were conducted on day 1 in order to assess the workplace background conditions in terms of the PNC, D_avg_, and LDSA of nanoscale airborne materials. Furthermore, two parallel sessions conducted at the beginning and at the end of the measurement’s campaign allowed to calculate the parameters of correlation useful to harmonize the instruments’ responses. For this purpose, all the instruments were positioned at the same time in the same location, e.g., in the center of the production laboratory. In particular, DM has been compared to the CPC signal for PNC corrections (Figure S1) and to the NSAM signal for LDSA corrections (Figure S2). The corresponding parameters of correlation lines (PNC_CPC_ = α∗PNC_DM_ + β, LDSA_NSAM_ = δ∗LDSA_DM_ + γ) have been obtained (Table S1). Furthermore, comparisons among two DMs have been conducted to set the instrument’s diffusion (I_diff_) and filter (I_filter_) stage current signals according to equations in Fierz et al. [46] and to calculate D_avg_ corrections (Figure S3). As a reference instrument, the DM that better correlates to CPC values has been chosen [47]. Instrument comparison results have been thoroughly described in Supplementary Materials. In the following text, the values corrected after the instrument comparison analysis are identified by an asterisk.

**Figure 3:**
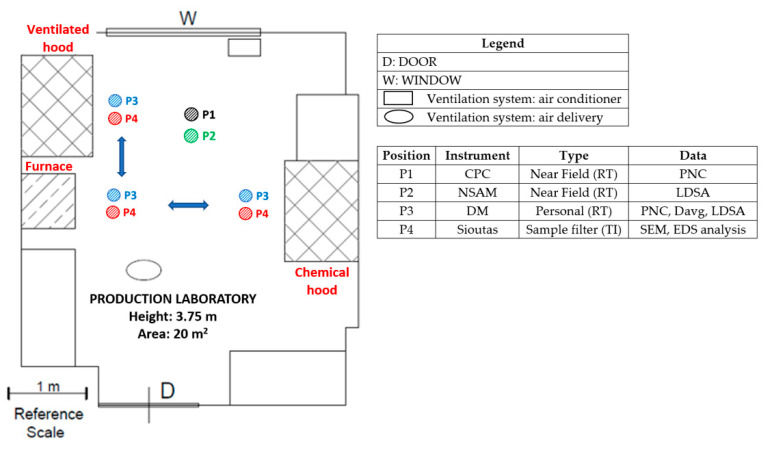
Instruments’ location and sampling points in the production laboratory.

Figure 3 represents a schematic plant of the production laboratory, showing the locations where the real-time instruments and the personal samplers were positioned during the production phases, and the corresponding instruments’ information. In particular, the worker’s exposure was measured in the personal breathing zone (PBZ), i.e., within a 0.3 m radius of worker’s nose and mouth, before and during the activities. The worker’s personal exposure was monitored by the DM-UF5 (P3) and by Sioutas personal impactor (P4), placed on the lab coat, with the sampling probes in its PBZ. During the activities, the worker moves to the different workstations to perform each phase, as showed by the arrows (Figure 3). In the same way, we measured the workplace exposure at the NF location (i.e., the position of another worker not directly involved in the process phases). The NF measurements were performed by using the CPC (P1) and the NSAM (P2). In the room located next to the production laboratory, FF background measurements were performed simultaneously with the production by using the second DM (DM-UF3).

## 3. Results and Discussion

### 3.1. Characterization of the Background Environment When No Production Occurred

Background mean values and standard deviations of PNC, D_avg_, and LDSA and the PNC significant values, referred to each day of measurement for all real-time sampling locations (NF, PBZ, and FF), are summarized in Table 3. It should be noted that during the background measurements, the PBZ position of DM-UF5 corresponds to the same NF positions of CPC and NSAM instruments.

Table 3 shows that the background conditions of the laboratory and the FF room slightly differ on day 2 compared to day 3. In particular, the PNC mean values (and the corresponding standard deviations) of day 2 are higher (about 2000 part/cm^3^) than the corresponding mean values measured on day 3. For this reason, the significant values on day 2 could represent the worst exposure scenario. However, on both days, the NF and PBZ mean PNC values are higher than the FF one, similarly to the situation reported in the previous study for GNPs liquid exfoliation laboratory [33]. With reference to the D_avg_ measurements, the mean NF value of about 55 nm is ~15 nm lower than the mean value in the FF room (~70 nm on day 2 and ~68 nm on day 3). Background differences between two subsequent days of measurements during graphene manufacturing in the same workplace were also reported by Lee et al. [28]. In the present case, since the differences are highlighted both in the lab and in the FF room, they are probably due to external factors such as different outdoor air features, weather conditions, temperature, relative humidity, etc.

Concerning the differences highlighted between NF/PBZ and FF background parameters on the same day (on day 2 and day 3), the FF room was chosen as representative of a workplace environment in which PNC levels are not influenced by the activity with NMs, though they may be dependent on the circumstances, e.g., room volume, wind direction, etc. [48]. In any case, the use of aspiration hoods inside the lab may contribute to reducing the D_avg_ compared to the FF room. Finally, although the two rooms have similar features, specific procedures for ventilation are in place in the lab compared to the FF room; e.g., the aspiration hoods are operating before, during, and after the activities based on the process requirements, the door opening is scheduled based on the specific process in place, and only when the aspiration hoods are turned on is the window opening allowed, and only after the end of the activities.

### 3.2. Real-Time Measurements

**Figure 4:**
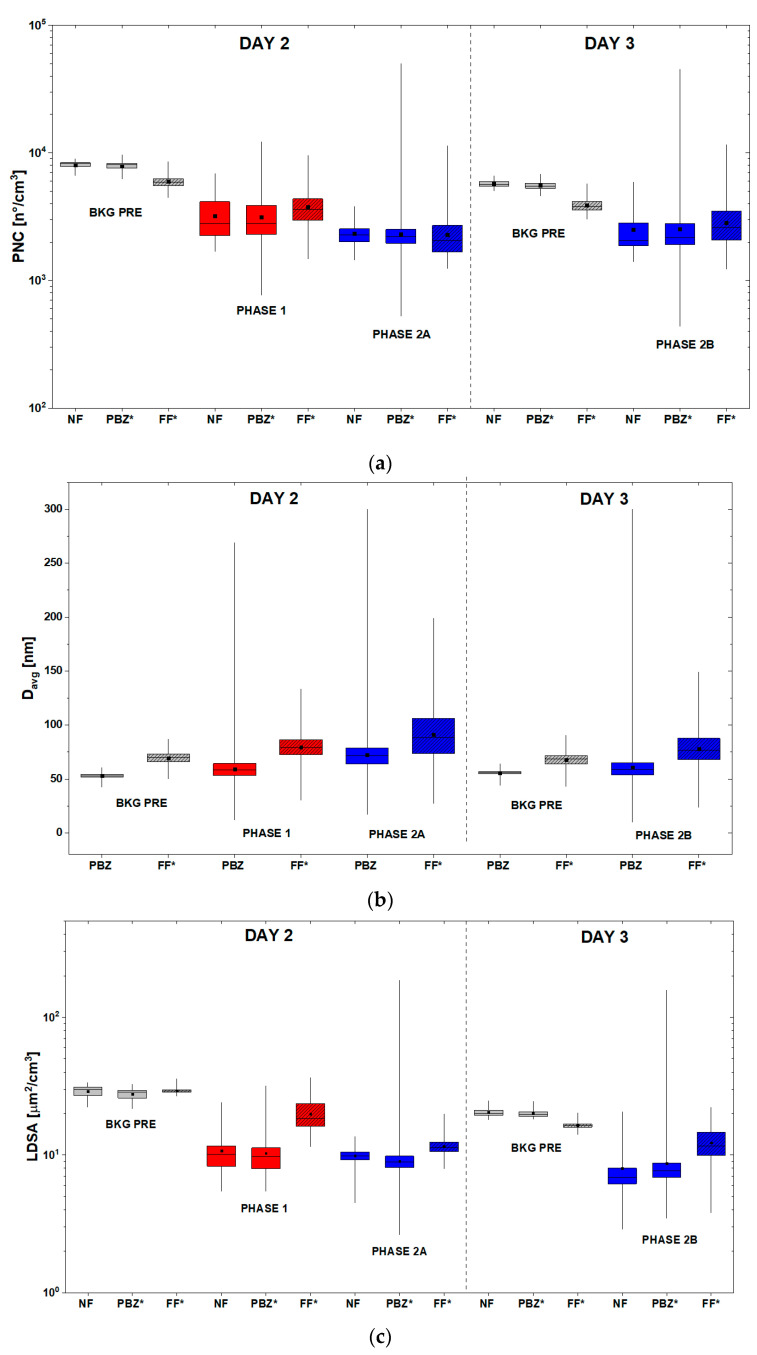
Box-plot diagrams of (**a**) PNC, (**b**) D_avg_, and (**c**) LDSA, measured before the activities (BKG PRE) and during the production phases of the two paints on day 2 (PHASE1, PHASE 2A) and day 3 (PHASE 2B) for all the corresponding real-time sampling locations (NF, PBZ and FF)). * values corrected after instrument comparison analysis.

The distribution parameters of PNC, D_avg_, and LDSA, which referred to both the background (BKG PRE) and the production (phase 1, phase 2A, phase 2B), are represented by the box-plot diagrams in Figure 4.

The PNC box-plots (Figure 4a) show that on day 2, the mean PNC values of the background NF (before starting the production) are quite high values if compared with those of phase 1 and phase 2A. A general decrease of the mean PNC during the measurements from BKG PRE to phase 2A, both in the laboratory and in the FF room, can be observed. In particular, in the PBZ, the PNC reached a maximum value of ~12,500 part/cm^3^ during phase 1, lower than the NF one but higher than the FF maximum value. During phase 2A, the PNC reached a maximum value of ~50,000 part/cm^3^, higher than both the NF and FF ones. The boxplots of day 3 show a similar decrease in the PNC mean values during the measurements, from bkg_NF_ to the production phase 2B. In particular, during phase 2B, the maximum PNC recorded value is comparable to that measured during phase 2A and it is equal to ~45,000 part/cm^3^.

LDSA values reported in Figure 4c confirm a relationship with PNC values. Indeed, as observed from the PNC box-plots, the LDSA mean values recorded before starting production are always higher than those measured during all the production phases (phase 1 and 2A of day 2 and phase 2B of day 3). On both days 2 and 3, the mean LDSA values in the production lab (NF and PBZ) are lower than those of the FF. However, during phase 2A and phase 2B in the PBZ position, LDSA reached maximum a value of ~200 µm^2^/cm^3^ on day 2 and of ~150 µm^2^/cm^3^ on day 3, both higher than the corresponding maximum values of NF and FF positions and higher than the values of the background before the production.

Box-plots of D_avg_ (Figure 4b) show that on day 2, before starting the production and during both the production phases 1 and 2A, the D_avg_ in the PBZ position was lower than in the FF room. Moreover, the interquartile range of D_avg_ during the whole measurements increased, both in the PBZ and in the FF positions. In particular, D_avg_ of PBZ during phase 2A reached the value of 73 nm, substantially higher than D_avg_ of the BKG before production (about 55 nm) and D_avg_ of phase 1 and 2B (about 59 nm). Looking at the whiskers of the boxplot which referred to the PBZ position, D_avg_ reached a maximum value of ~275 nm during phase 1 and another maximum value of ~300 nm (out of scale of DM device) during phase 2A. Similar situation of phases 1 and 2A can be observed during phase 2B, where D_avg_ in the PBZ (56 nm before the production and 60 nm during the production) was lower than in the FF room, both before (68 nm) and during the work phase 2B (77 nm). However, also during this phase, D_avg_ reached a maximum value out of the scale of the measurement device.

**Figure 5:**
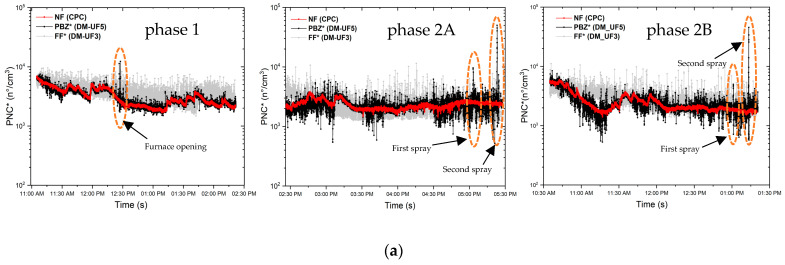

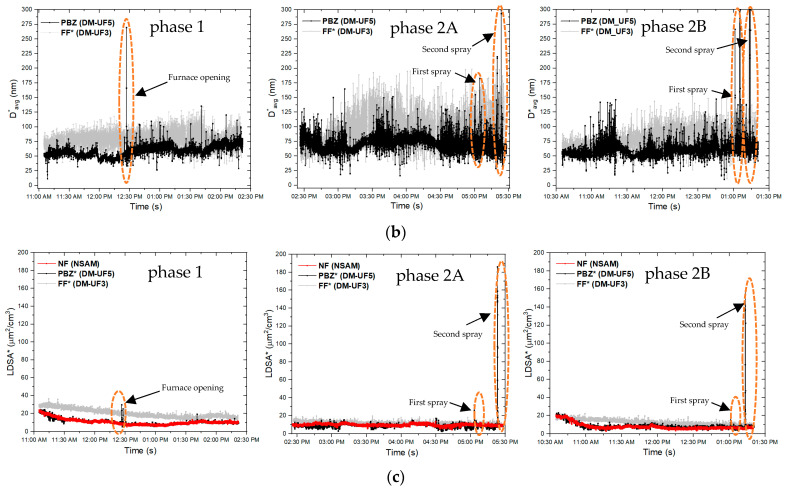
Time series of (**a**) PNC, (**b**) D_avg_, and (**c**) LDSA of the real-time measurements performed during the production phases 1, 2a, and 2b. * values corrected after instrument comparison analysis.

Figure 5 reports the time series of PNC, D_avg_, and LDSA referring to the real-time measurements performed in the production lab and in the FF room on both days of production.

From the time series, it can be noted that the PNC decreased during the whole production process, as already highlighted by Figure 4a, both inside and outside the laboratory and on both days of measurements.

During phase 1, in the PBZ and NF positions inside the lab, the PNC always shows average values lower than those of the FF position, except for the peak recorded at 12:27 p.m. in the PBZ, equal to 12,300 part/cm^3^, which exceeds both the PNCs of the BKG FF and the PNC significant value, corresponding to 9800 part/cm^3^. This peak arises while opening the furnace to introduce the GNP/acetone solution in order to obtain a dry GNP powder after solvent evaporation. LDSA decreases over time during the whole production, following a similar trend to the PNC, with values inside the laboratory lower than those of the BKG FF. In particular, in correspondence of the PNC peak during phase 1, LDSA reaches a maximum value of 30 µm^2^/cm^3^, which exceeds the LDSA of the BKG FF but is comparable with the mean values measured before starting the production (see Table 3). The D_avg_ during phase 1 increases and it reaches a peak value of ~275 nm in the PBZ in correspondence of the furnace opening at 12:27 p.m., which is a very high value if compared with the average value before and after the operation, approximately equal to 55 nm. It is worth noting that during the GNP thermal expansion process, the decrease of D_avg_ in correspondence to the muffle furnace opening was associated with the emission of other non-carbonaceous materials such as expansion agents of graphite intercalated compounds, NPs constituting the inner walls of the furnace (i.e., alumina), or even “worm-like” expanded graphite fragments detached from the main structure with dimensions of a few nanometers [33]. On the contrary, in this case, the simultaneous increase of D_avg_, PNC, and LDSA when the furnace was opened may be clearly associated to the emission of large particles, not necessarily due to the sole contribution of GNPs emission.

During phase 2A, the BKG FF PNC continues to decrease until reaching the same mean values of the laboratory. Two PNC peaks were recorded in the PBZ, with corresponding D_avg_ and LDSA peaks: the first one at 5:04 p.m. is equal to 10,000 part/cm^3^ and the second one at 5:23 p.m. is equal to 49,000 part/cm^3^. This last value widely exceeds the PNC BKG significant value. When the first PNC peak occurs, D_avg_ reaches a maximum value of 175 nm and LDSA is equal to 30 µm^2^/cm^3^. Meanwhile, when the second PNC peak occurs, the D_avg_ reaches a higher value of 300 nm and the LDSA a value equal to 189 µm^2^/cm^3^. As we expected, these two peaks took place in correspondence with each spray coating deposition of the paint loaded with 3.5 %wt of GNPs.

The other two PNC peaks occur in the worker’s PBZ during phase 2B, corresponding to the other two spray depositions of the paint loaded with higher GNP concentration. The first peak takes place at 01:02 p.m., reaching a PNC value of ~5000 part/cm^3^, an average diameter of 300 nm, and a maximum LDSA value of 20 µm^2^/cm^3^. The second PNC peak takes place at 01:14 p.m., reaching a value of ~45,000 part/cm^3^, with a corresponding D_avg_ of 300 nm and LDSA of 157 µm^2^/cm^3^.

It should be noted that the first PNC peaks of both phases 2A (at 05:04 p.m.) and 2B (at 01:02 p.m.), corresponding to the spray depositions performed with the smaller nozzle (diameter of 0.8 mm), are lower than the second (at 05:17 p.m. for phase 2A and at 01:14 p.m. for phase 2B) peaks performed using the airbrush equipped with the bigger nozzle (diameter of 1.2 mm). In particular, during the first spraying, the PNC reach two different maximum values, equal to ~10,000 part/cm^3^ (phase 2A) and ~5000 part/cm^3^ (phase 2B), respectively, while during the second spraying, the PNC peaks reach ~50,000 part/cm^3^ in both cases (phase 2A and phase 2B), independently of the amount of nanofiller inside the paint. When the nanostructured paint is sprayed with smaller nozzle, the deposition is influenced by the amount of nanofiller inside the paint, thus justifying the choice already adopted at the design stage of the manufacturing process to use the bigger nozzle for the spray deposition of the paint loaded with GNPs concentrations higher than 5 %wt.

**Figure 6:**
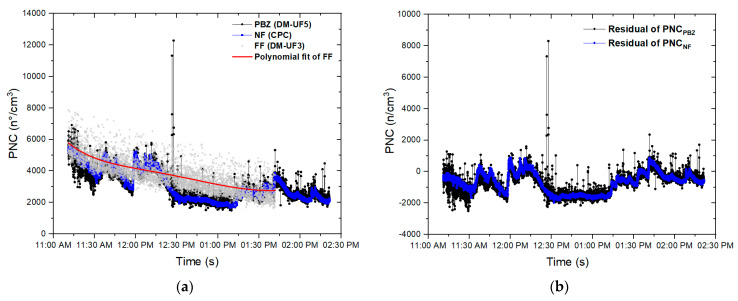
(**a**) Polynomial fitting and (**b**) residual curves of the PNC time series during phase 1.

The PNC time series of phase 1 shows that PNC inside the laboratory is strongly correlated to the background FF. In fact, the average PNC in the FF room tends to decrease over time during the entire production phase with a trend that is also reflected within the laboratory. This behavior is probably due to a change in the outdoor conditions which influenced the whole environment of the facility in which the laboratory is located. Consequently, in order to remove the background contribution, we proceeded to subtract the polynomial fit curve of FF from the time series of the PNC measured inside the laboratory during the production process (NF and PBZ) as shown in Figure 6a. The obtained residual curve is reported in Figure 6b and it highlights that PNC increases during the sonication phase that takes place from 11:28 a.m. to 12:08 p.m. and from 12:28 p.m. to 02:25 p.m. during the acetone evaporation in furnace.

In summary, the PNC figures, after subtracting the background, resulted lower than the significant value during the whole phase 1, reporting no task-related NMs emissions. Otherwise, phase 2A and phase 2B showed peaks exceeding 4–5 times the significant value but lower than the proposed NRV_15-min TWA_ [23] after subtracting the background.

In the present study, instruments for exposure assessment have been used according to the features recommended by OECD Tier 2 such as, easy to use, battery operated, handheld, and able to deliver a useful dataset to estimate exposure levels. Otherwise, accuracy may be lower than the massive equipment [49]. The affordability of measurements may be influenced by differences between instruments used (i.e., CPC and DCs) related to principle of operation, size range, and upper/lower limits of detection and operating conditions when the measurement is carried out, e.g., use of sampling tubes or aerosol that do not match with the calibration fluid [50]. In order to overcome some of these drawbacks, Tygon^TM^ sampling lines were used for DM measurements to minimize the losses according to Asbach et al. [51]. Furthermore, we proposed an instruments comparison session in the workplace before and after the sampling campaign, to identify the correction factors, and align the PNC, D_avg_, and LDSA values measured by different devices (as described in Supplementary Materials). The CPC was used as a reference instrument for PNC comparison [49]. The DM that showed high comparability with CPC was used as a reference instrument for D_avg_ calibration [47]. The performance of the NSAM monitor was considered very robust in its use as a reference instrument for LDSA values [52]. Although the inter-comparison conducted for the room air may be relevant for the background levels identification, uncertainties still remain when the GNPs emissions during the production process are measured. The spherical approximation of particles on which DC measurements (DM and NSAM) are based may induce further limitations also to D_avg_ and LDSA measured levels when the emissions are related to bi-dimensional NMs such as GNPs.

### 3.3. SEM and EDS Analysis on Sampled Materials

SEM investigations and EDS analysis have been conducted on the materials collected by the filters of Sioutas personal impactor in order to match and support the results of the real-time measurements that showed potential workers’ exposure to GNPs during the spray deposition of the nanostructured paint.

As a result of samplings, GNPs were found in all the filters of the Sioutas stages, independently of their sizes. This is probably due to the unusual aerodynamic property of such plate-like particles [18] and, as a consequence, to the fact that the cascade impactor’s working principle is based on aerodynamic equivalent particle sizes [53].

**Figure 7:**
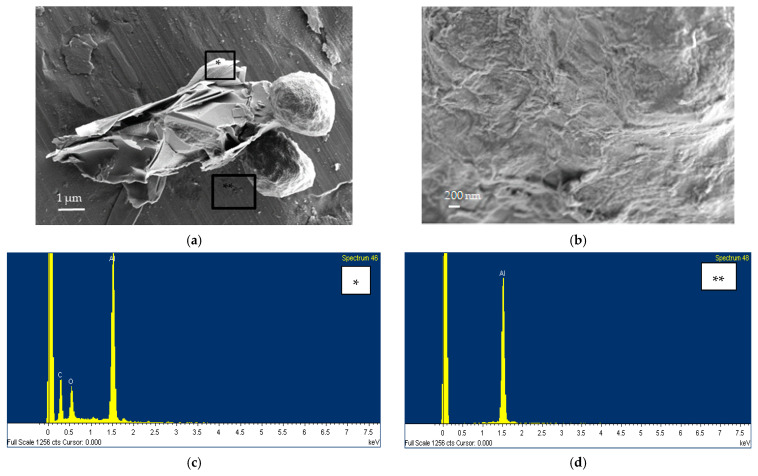
SEM image (**a**) and EDS spectra in the points signed as * (**c**) and ** (**d**) on the materials collected by Sioutas (filter A); SEM image of a trial sample of the spray-coated water-based PU paint (**b**).

Figure 7a shows the results of SEM investigations conducted on the filter stage A of the personal impactor, where aggregated nanostructures, characterized by a few layers of thin flakes with irregular geometry and shapes, were found. It is possible to notice their particular aggregation, forming a folded-like shape. The results of EDS analysis reported in Figure 7c shows the C and O signals came from the carbon-based GNPs structures and the Al signal is due to the aluminum sampling filter (Figure 7d). These materials are linked to spherical-shaped materials with a diameter of a few microns and a roughened surface, very similar to the surface of the water-based polyurethane paint, whose SEM image is reported in Figure 7b.

**Figure 8:**
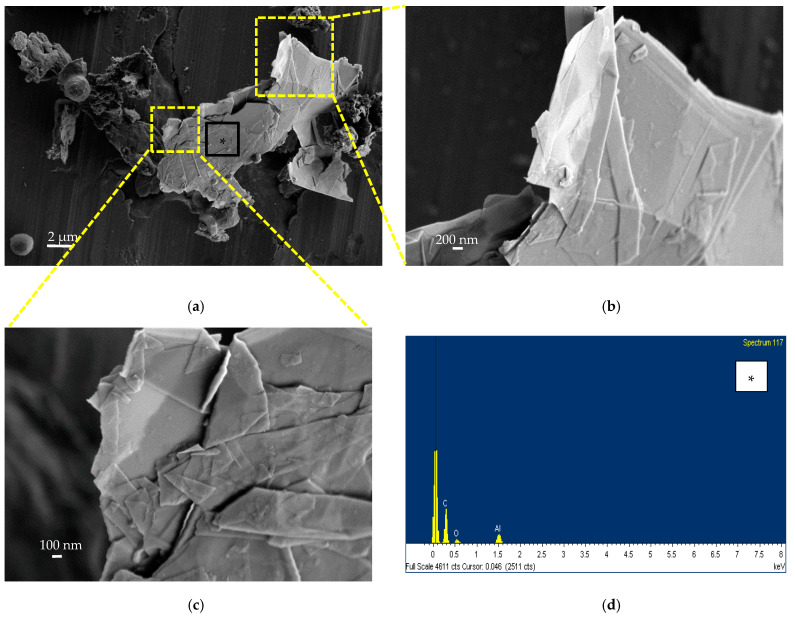
(**a**–**c**) SEM images and (**d**) EDS analysis on the airborne sampled materials collected on filter B, corresponding to the point signed as * in (**a**).

We also found the same nanostructures, linked to airborne particulate matter, on the filter stage B (see Figure 8a). In this case, the whole geometry of the aggregated nanostructures has a more flattened shape with flakes of different lateral sizes (Figure 8b,c). The EDS spectrum reported in Figure 8d shows the carbon-based structure of the collected nanomaterial.

According to the high-resolution SEM images (Figure 7 and Figure 8), both planar and folded GNP-like structures could be observed. The folded structure may be attributed to the large lateral-size-to-thickness ratio and out of plane flexibility of GNPs. The folded configuration could also be induced by the mechanical stimulation during the spray coating procedure [54].

Such findings, compared to the morphological evidence by other similar studies on GNPs [29], also highlight GNP-like materials with lateral dimension of a few microns included in large carbon-based platelets structures.

## 4. Conclusions

In the present study, measurements of potential emissions of airborne GNPs were carried out during the manufacturing process of water-based PU/GNP paint for producing multifunctional composite materials for both SHM and EMI applications. On the basis of the OECD methodology tier 2, high-resolution real-time measurements and time-integrated samplings were performed in the workplace during the production phases and the deposition by spray-coating technique of two types of paints: the first one loaded with 3.5 %wt of GNPs; and the second one loaded with 7 %wt of GNPs with respect to the mass of the neat PU paint. Off-line analysis through SEM EDS was carried out to characterize the airborne particles collected in the workers’ PBZ by the Sioutas impactor.

The major findings of the study are summarized as follows.

In general, during the production, the PNC, D_avg_, and LDSA inside the laboratory show median values lower than those of the BKG FF (and also of the BKG NF before starting the activities), leading us to relate such a figure to the effectiveness of lab ventilation hoods in removing airborne particles.

In a large part of the production process, the background contribution heavily influenced the PNCs inside the laboratory, as evidenced by the comparison between the PNC curves of phase 1 referring to PBZ and NF inside the lab and the PNC curve of the FF room. After the removal of the background influence, it was possible to highlight PNC’s increase in correspondence to the sonication and the evaporation of the acetone in the furnace; it was lower than the PNC’s significant value.

During phase 1, PNC and LDSA time series show higher levels in the FF room than in the operator’s PBZ and in the NF laboratory sampling points, except for the peak at 12:27 p.m.: it exceeded the PNC significant value, with a contemporarily increase of the D_avg_, which was aroused when opening the furnace to place the GNP/acetone suspension inside it for the solvent evaporation phase.

During phase 2A and during phase 2B, two PNC (and LDSA) peaks, exceeding the PNC significant value, were observed in the worker’s PBZ in correspondence with the single spraying activity. Such values are lower than the NRV_15 min TWA_ proposed in the literature. Furthermore, when the PNC peaks occur, an increase of the D_avg_ was observed, reaching maximum values out of the scale of the measurement device, indicating the presence of particles with average size larger than 300 nm.

The findings of SEM and EDS analyses confirmed the evidence obtained from real-time measurements which stated that on the filters of the personal impactor worn by the worker during the production, few-layers of carbon-based thin flakes with irregular geometry and shapes were found. These structures, with a lateral size of a few microns, are characterized by two different types of geometry: a folded-like geometry and a flatter one. The folded-like geometry can probably be ascribable to the out of plane flexibility and high aspect ratio (large lateral size to thickness ratio) of GNPs. It may also be attributable to mechanical stimulation during the spray coating procedure.

In conclusion, PNC and LDSA results highlighted a potential GNPs release during the spray-deposition of the nanostructured coating near the operator who performs the deposition, representing the worst potential exposure conditions of the production process. However, the possible release in the work environment occurs in a restricted area, thus not involving other workers who may eventually be present in the production laboratory, since the instrument located in the NF sampling point (CPC) has not recorded any PNC peak. Furthermore, the ventilated hood, which aspirates the exceeding particles produced during the spray coating almost instantaneously, guarantees a rapid reduction of PNC, which almost instantly returns to safety levels, limiting the exposure time.

Additionally, GNPs are frequently linked to other structures of airborne material of micrometric size. Therefore, for this size of airborne particles, the filters of the full-face respirators worn by worker during the activities maintain their efficiency, certified by the UNI EN 14387 standard for the class of dust filters P3, with penetration requirements tested according to the UNI EN 13274-7 standard. However, in this case, the exposure may occur for short periods but with great spillage amounts; thus, according to the hierarchy of risk management measures, engineering controls and collective protection measures may be preferred. It is therefore recommended to perform the spray coating in an enclosed system or by using a glove box or, alternatively, by introducing a remotely controlled automated device.

Finally, the outcomes provide important information in order to anticipate the risks for workers who produce and handle GNPs for the development of innovative graphene-based nanocomposites, and can be used to improve future design. Although the emission parameters alone do not predict the effective worker exposure, the proposed measurement strategy, integrated in a PtD approach for the design of novel engineered nanocomposite materials, represents a promising, good practice for R&D laboratories where any NMs-based innovation is undertaken. Furthermore, it may improve and facilitate the technology scale-up at the industrial level, at which risks reduction strategies can be more easily identified and implemented thanks to the lessons learned during the early stages of the development chain.

## Figures and Tables

**Table 1:** Materials used for the production of the PU/GNP paints.

Material	Acetone [mL]	GNPs [g]	Pu Paint and Hardener [g]	DI Water [mL]
Paint type A	150	1.75	62.5	10
Paint type B	150	4	62.5	40

**Table 2:** Timesheet of measurements and sampling conducting during the production of paint type A and paint type B.

Day	Time	Measurements
DAY 1 Instruments setup	12:15 p.m.–01:35 p.m.	Instruments comparison
DAY 2 Production: phase 1 and phase 2A (paint type A)	10:50 a.m.–05:25 p.m.	Background FF
	10:50 a.m.–11:05 a.m.	Background NF
	11:10 a.m.–02:20 p.m.	Production: phase 1
	02:25 p.m.–05:25 p.m.	Production: phases 2A
DAY 3 Production: phase 2B (paint type B) Instruments setup	10:20 a.m.–01:20 p.m.	Background FF
	10:20 a.m.–10:35 a.m.	Background NF
	10:40 a.m.–01:20 p.m.	Production: phases 2B
	02:05 p.m.–02:45 p.m.	Instruments comparison

**Table 3:** Background values of PNC, D_avg_, and LDSA before the production activities in Day 2 and Day 3, by sampling position (NF, PBZ, or FF) and measurement instrument (CPC, DM, or NSAM).

		DAY 2			DAY 3		
		NF	PBZ *	FF *	NF	PBZ *	FF *
PNC (part/cm^3^)		CPC	DM	DM	CPC	DM	DM
	Mean	8000	7800	5900	5700	5500	3900
	σ	600	700	600	300	400	500
	Significant Value	9800	9900	7700	6600	6700	5400
D_avg_ (nm)			DM	DM		DM	DM
	Mean	-	53	70	-	56	68
	σ	-	2	6	-	2	6
LDSA (μm^2^/cm^3^)		NSAM	DM	DM	NSAM	DM	DM
	Mean	29	28	29	20	20	16
	σ	3	3	1	2	1	1

* values corrected after instrument comparison analysis.

## Data Availability

The data presented in this study are available on request from the corresponding author.

## Supplementary Materials

The following supporting information can be downloaded at: https://www.mdpi.com/article/10.3390/nano13081378/s1, Figure S1: UF3-CPC (a) and UF5-CPC (b) PNC scatter plots and linear fits.; Figure S2: UF3-NSAM (a) and UF5-NSAM (b) LDSA scatter plots and linear fits; Figure S3: DM-UF3–DM-UF5 Idiff (a) and Ifilter (b) scatter plots and linear fits. Table S1: Correlation’s parameters of PNC (a), D_avg_ (b) and LDSA (c).
