# Supplementary material for: Occupational Exposure during the Production and the Spray Deposition of Graphene Nanoplatelets-Based Polymeric Coatings
Source: Nanomaterials (Basel). 2023 Apr 15;13(8):1378. doi: 10.3390/nano13081378 (PMC10142999; doi:10.3390/nano13081378)
Supplement: Supplementary file 1 [file nanomaterials-13-01378-s001.zip › nanomaterials-2237280-supplementary.pdf]

Supplementary Materials

In order to harmonize the instrumet measurements and to make a reliable comparison between the values of PNC, D_{avg} and LDSA measured during the production process and the corresponding values of the background, we made a comparison of the different real time instruments response in real operating conditions. At the end and at the beginning of the production process monitoring, two simultaneous measurement sessions were performed inside the production laboratory, by using all the available instruments in operating conditions. The room air with PNC varying from 1000 to 6000 part/cm^3 was used as sampling athposphere. The CPC as been used as reference instrument for the PNC calibration, the DM-UF5 has been used as reference instrument for D_{avg} calibration of DM-UF3, and the NSAM has been used as reference instrument for the LDSA calibration. The results of this comparison are reported respectively in the following Figure S1, S2 and S3. The corresponding parameters of correlation lines have been obtained for PNC, D_{avg} and LDSA values and summarized in the following Table S1.

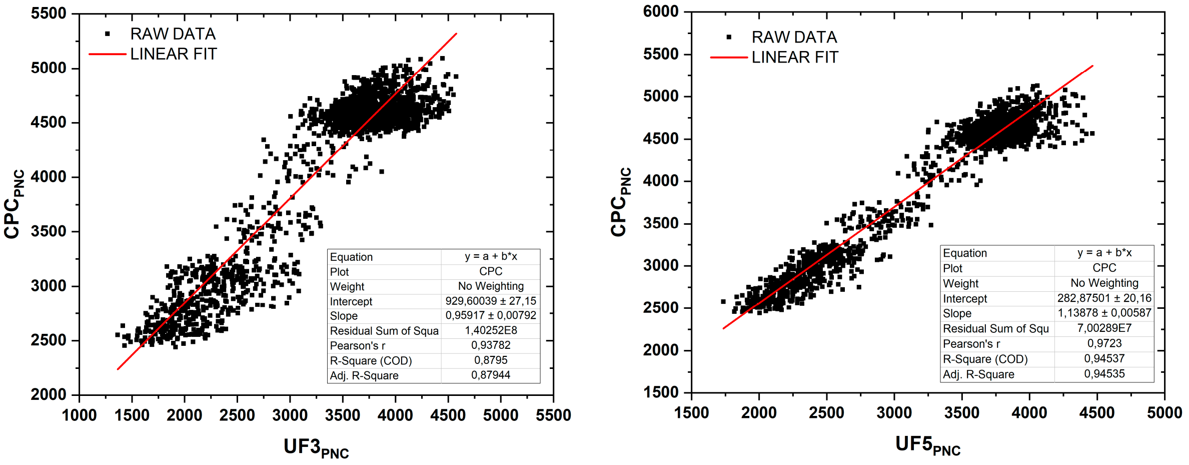

(a) *(b)*

Figure S1: UF3 - CPC (a) and UF5 - CPC (b) PNC scatter plots and linear fits.

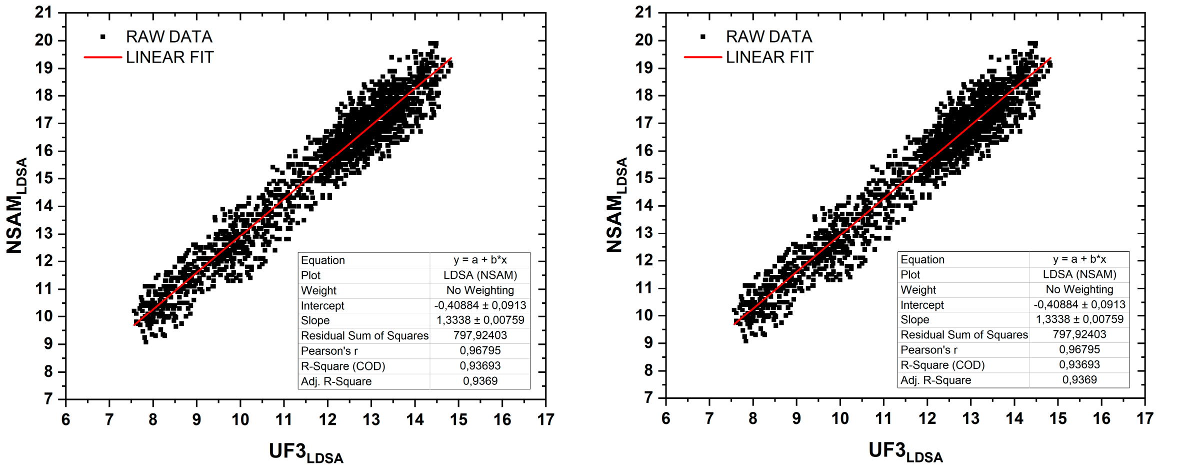

(a) *(b)*

Figure S2. UF3 - NSAM (a) and UF5-NSAM (b) LDSA scatter plots and linear fits.

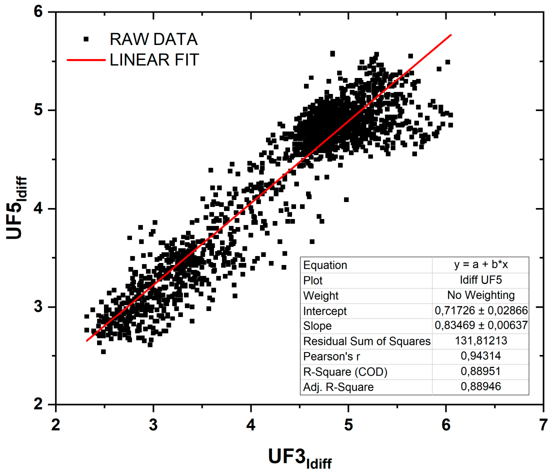

(a)

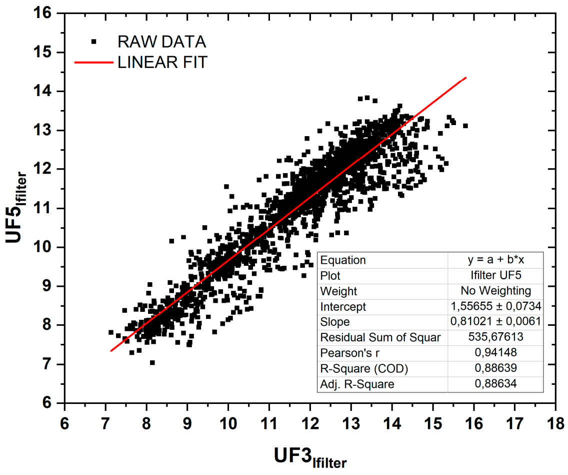

(b)

Figure S3 DM-UF3 – DM-UF5 I_{diff} (a) and I_{filter} (b) scatter plots and linear fits.

Table S1: Correlation's parameters of PNC (a), D_{avg} (b) and LDSA (c)

	PNC			
	α	β	R^2	Pearson's coeff.
CPC/UF3	0.95917	929.60039	0.8795	0.93782
CPC/UF5	1.13878	282.87501	0.94537	0.9723

(a)

	I_{filter} (D_{avg})			
	α'	β'	R^2	Pearson's coeff.
UF5/UF3	0.81021	1.55655	0.88639	0.94148

	I_{diff} (D_{avg})			
	α''	β''	R^2	Pearson's coeff.
UF5/UF3	0.83469	0.71726	0.88951	0.94314

(b)

	LDSA			
	δ	γ	R^2	Pearson's coeff.
NSAM/UF3	1.3338	-0.40884	0.93693	0.96795
NSAM/UF5	1.40519	-1.66698	0.95852	0.97904

(c)
